# Open Issues in the Transition from NAFLD to MAFLD: The Experience of the Plinio Study

**DOI:** 10.3390/ijerph18178993

**Published:** 2021-08-26

**Authors:** Francesco Baratta, Domenico Ferro, Daniele Pastori, Alessandra Colantoni, Nicholas Cocomello, Mattia Coronati, Francesco Angelico, Maria Del Ben

**Affiliations:** 1 I Clinica Medica, Department of Clinical Internal, Anesthesiological and Cardiovascular Sciences, Sapienza University, 00161 Rome, Italy; francesco.baratta@uniroma1.it (F.B.); domenico.ferro@uniroma1.it (D.F.); alessandra.colantoni@uniroma1.it (A.C.); nicholas.cocomello@uniroma1.it (N.C.); mattia.coronati@gmail.com (M.C.); maria.delben@uniroma1.it (M.D.B.); 2 Emergency Medicine Unit, Department of Clinical Internal, Anesthesiological and Cardiovascular Sciences, Sapienza University, 00161 Rome, Italy; 3 Department of Public Health and Infectious Diseases, Sapienza University, 00161 Rome, Italy; francesco.angelico@uniroma1.it

**Keywords:** non-alcoholic fatty liver disease, metabolic associated fatty liver disease, non-alcoholic steatohepatitis, obesity, diabetes, metabolic syndrome

## Abstract

Metabolic associated fatty liver diseases (MAFLD) definition was proposed to identify fatty liver condition associated to metabolic disorders and to replace non-alcoholic fatty liver disease (NAFLD). We aimed to explore the effect of the application of the new MAFLD criteria on a pre-existing cohort of NAFLD patients. The consequences of the reclassification were investigated by applying the MAFLD criteria to a prospective cohort (The Plinio Study) of dysmetabolic patients examined for the presence of NAFLD. In the Plinio cohort, 795 patients had NAFLD and 767 of them (96.5%) were reclassified as MAFLD patients. Out of these, 94.9% had overweight/obesity or diabetes, while the remaining were lean and had metabolic dysregulation defined by the presence of at least two metabolic risk abnormalities. By contrast, 3.5% of the NAFLD patients were reclassified as no-MAFLD due to the absence of overweight/obesity, diabetes, or metabolic risk abnormalities. The only significant difference between the NAFLD and MAFLD groups was the higher prevalence of subjects with BMI ≥ 25 kg/m^2^ in the latter (88.6% vs. 92%; *p* = 0.018). In the cohort, 68 subjects were defined as “lean NAFLD”. Of these, 40 were reclassified as MAFLD and 28 as no-MAFLD. In conclusion, when applying MAFLD criteria to the Plinio cohort, there is a substantial overlap between NAFLD and MAFLD diagnosis. However, some specific subgroups of patients, such as those currently defined as lean NAFLD, were excluded by the new MAFLD definition.

## 1. Introduction

Non-alcoholic steatohepatitis (NASH) definition was introduced in 1980 to describe an alcoholic-like hepatitis in patients without a history of alcohol abuse or hepatotropic viruses infection, mainly in obese patients [1]. Afterward, the term of non-alcoholic fatty liver disease (NAFLD) was firstly used to describe a less severe steatosis condition [2,3] and then to define the wide spectrum of liver conditions associated to steatosis, ranging from simple fatty liver to cirrhosis [4].

Since the beginning, insulin resistance was proposed as a major NAFLD risk factor [5] and authors have long debated whether NAFLD was the hepatic feature of the metabolic syndrome (MetS) [2]. Based on this assumption in 1998, C.P. Day and O.F. James drafted the “Two-hits hypothesis”, defining the insulin resistance as the “primum movens” in the NAFLD pathogenesis and the oxidative stress as the second hit in the progression from simple steatosis to NASH [6,7]. Over the years, a more complex pathogenesis of NAFLD has become evident, leading to the so-called “multiple hits hypothesis” [8]. Based on this theory, many factors may contribute to NAFLD onset and progression, including diet [9], oxidative stress [10], gut microbiota [11], and genetic factors [12,13].

Nowadays, the definition of NAFLD includes different clinical phenotypes of non-alcoholic and non-viral steatosis, which may have different clinical outcomes, including both obese patients with metabolic NAFLD and lean genetic ones, the latter characterized by the lack of insulin resistance [12,14,15].

A recent expert consensus underlined that NAFLD remains an exclusion diagnosis and claimed the need for more accurate diagnostic criteria [16], more closely connected with the pathogenic processes. The effort to identify a more homogeneous population of patients aims to drive a more effective drug research for this condition [16]. For this reason, in 2020, the definition of “metabolic dysfunction-associated fatty liver disease—MAFLD” has been firstly proposed to identify fatty liver condition associated to metabolic disorders [17]. Metabolic disorders include diabetes, overweight, or a combination of two out of five minor cardio-metabolic abnormalities (high waist circumference, low HDL, high triglycerides, impaired fasting glucose, high blood pressure, high C-reactive protein (CRP), insulin resistance). Alcohol abuse or viral hepatitis do not exclude the MAFLD diagnosis. In addition, the authors optionally suggested the use of biochemical scores (e.g., fatty liver index) to identify fatty liver in large population studies.

This new definition has been regarded with cautious from many authors concerned about the risk of a premature change in terminology [18]. The main issue relates to the fact that the “MAFLD” definition does not solve many ambiguities inherent to the NAFLD classification, does not properly consider genetic steatosis, and does not lead to a better risk stratification in patients [18].

The effect of applying this new MAFLD definition to the already-existing cohorts of patients classified as NAFLD is still unclear.

The aim of this study was to evaluate the reclassification effect produced by the new MAFLD criteria application on a wide cohort of consecutive subjects diagnosed with NAFLD.

## 2. Materials and Methods

The Plinio Study (Progression of Liver Damage and Cardiometabolic Disorders in Non-alcoholic Fatty Liver disease: An Observational Cohort study. ClinicalTrials.gov Identifier: NCT04036357) is a cohort study of consecutive outpatients referring to the Day Service of Internal Medicine and Metabolic Diseases of the Department of Internal Medicine of Sapienza University of Rome for the management of cardio-metabolic risk factors. All patients had at least one out of the following cardio-metabolic diseases: arterial hypertension, overweight/obesity (BMI ≥ 25 kg/m^2^), type 2 diabetes, dyslipidemia, atrial fibrillation (AF), and metabolic syndrome (MetS). Exclusion criteria were: average daily consumption of alcohol >20 g in women and of >30 g in men [5], excessive drinking and alcohol use were further confirmed by the use of Alcohol Use Disorders Identification Test, AUDIT [19], presence of hepatitis B surface antigen and antibody to hepatitis C virus, positive tests for autoimmune hepatitis, other chronic liver diseases, diagnosis of oncological diseases and concomitant therapy with drugs known to promote liver steatosis (e.g., amiodarone), other chronic infectious or autoimmune disease, and clinical, biochemical, or ultrasonography (US) signs of cirrhosis or portal hypertension [20].

At the first visit, all patients underwent a complete clinical and biochemical diagnostic work-up, including routine clinical and biochemical evaluations. Anthropometric data (i.e., waist circumference and body mass index, BMI) and information on concomitant treatment and co-morbidities were registered. Cardiovascular and metabolic risk factors were defined according to international guidelines [21,22,23,24,25]. Liver US scanning was performed to assess the presence of steatosis. All US were performed by the same operator who was blinded to laboratory values using a GE Vivid S6 apparatus equipped with a convex 3.5 MHz probe. Severity of liver steatosis was defined according to a Hamaguchi score [26].

### 2.1. MAFLD Diagnosis

In the 987 Plinio patients included in the present analysis, aged 18–88 years, MAFLD diagnosis was defined by the presence of liver steatosis at ultrasound or fatty liver index (FLI) > 60 and at least one of the following conditions: overweight/obesity, diabetes, and metabolic dysregulation [17]. Metabolic dysregulation was defined by the presence of two or more of the following metabolic risk alterations: (1) Waist circumference ≥102 in men and 88 cm in women; (2) Blood pressure ≥130/85 mmHg or specific drug treatment; (3) TG ≥150 mg/dL or specific drug treatment; (4) HDL-C <40 mg/dL for men and <50 mg/dL for women; (5) Impaired fasting glucose (fasting glucose levels 100 to 125 mg/dL) or impaired glucose tolerance (glucose levels 140 to 199 mg/dL 2-h post-load) or HbA1c 5.7% to 6.4%; (6) Homeostasis model assessment-insulin resistance (HOMA-IR) score ≥2.5. Patients who did not meet the above diagnostic criteria were classified as non-MAFLD. Plasma high-sensitivity C-reactive protein, although an additional metabolic risk alteration, was not available in our dataset. FLI was calculated as previously reported [27].

Based on the above diagnostic criteria, and on the agreement between the definitions of NAFLD and MAFLD, patients were classified in the following four groups: Group 1—NAFLD/MAFLD; group 2—NAFLD/no-MAFLD; group 3—no-NAFLD/no-MAFLD; group 4—no-NAFLD/MAFLD.

FIB-4 and APRI scores were calculated to predict significant fibrosis [28]. The FIB-4 index was calculated using age, serum levels of AST, ALT, and platelet count, as previously described [29]. The APRI score was calculated as [(AST/ULN AST) ×100]/Platelet count [30].

### 2.2. Statistical Analysis

Categorical variables were reported as counts (percentages). The normal distribution of parameters was assessed by a Kolmogorov–Smirnov test. Normal variables were expressed as mean ± standard deviation, non-normal variables as median [IQR]. Independence of categorical variables was tested with the χ2 test. Student’s t and ANOVA tests were used to compare means. Mann–Whitney and Kruskal–Wallis tests were used to compare medians.

At first, a descriptive analysis according to the diagnosis of NAFLD and MAFLD was performed. Then, a comparison was performed between groups defined using both the original categories (NAFLD/no-NAFLD) and the target categories (MAFLD/no-MAFLD). We identified four groups: (1) NAFLD patients reclassified as MAFLD (NAFLD to MAFLD), (2) NAFLD patients not meeting MAFLD criteria (NAFLD to no-MAFLD), (3) no-NAFLD patients reclassified as MAFLD (no-NAFLD to MAFLD), and (4) patients no-NAFLD not meeting MAFLD criteria (no-NAFLD to no-MAFLD),

All *p* values < 0.05 were considered statistically significant. All analyses were performed with IBM SPSS 25.0 (IBM, Armonk, New York 10504-1722, NY, USA).

## 3. Results

Among the 987 patients enrolled in the “Plinio study”, 795 had NAFLD (80.5%) and 192 were no-NAFLD (19.5%).

Table 1 shows the clinical and biochemical characteristics of patients according to the presence or absence of NAFLD and MAFLD diagnosis. There was a substantial overlap in clinical and biochemical features regardless the definition used; the only statistically significant difference between NAFLD and MAFLD groups was the higher prevalence of subjects with a BMI > 25 kg/m^2^ (88.6% vs. 92.0%, *p* = 0.018) in the latter group.

### Reclassification Groups

**Figure 1:**
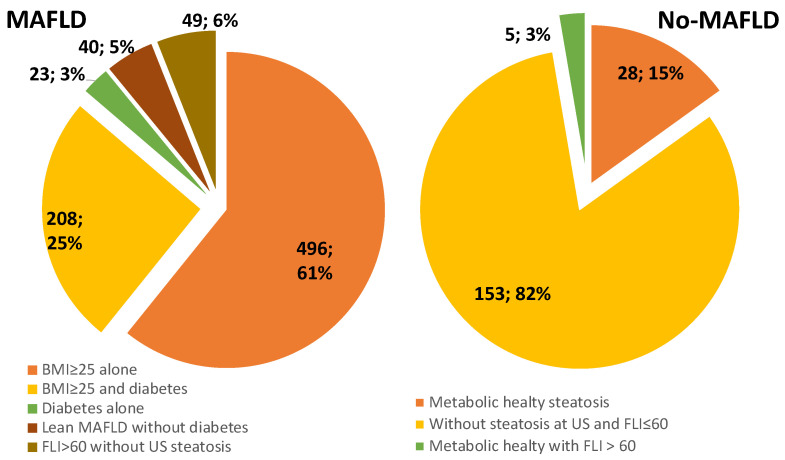
Diagnostic criteria for reclassification of PLINIO patients to MAFLD and No-MAFLD.

Figure 1 summarizes diagnostic criteria for the reclassification of PLINIO patients to MAFLD and no-MAFLD.

**Figure 2:**
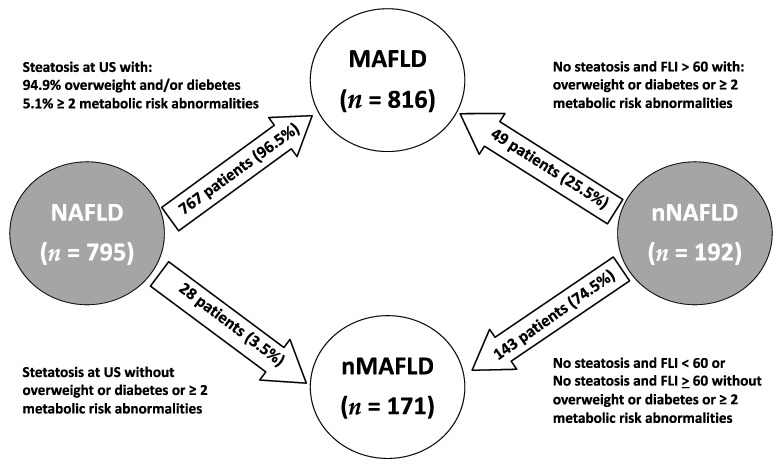
Reclassification of Plinio patients to MAFLD and no-MAFLD.

As reported in Figure 2, 767 (96.5%) NAFLD patients were diagnosed with MAFLD, based on the new diagnostic criteria. Out of these, 94.9% had overweight/obesity or diabetes, while the remaining were lean and had metabolic dysregulation defined by the presence of at least two metabolic risk abnormalities (lean MAFLD). By contrast, 28 (3.5%) out of the 795 NAFLD patients, despite US evidence of fatty liver on ultrasonography, were reclassified as no-MAFLD due to the absence of overweight/obesity, diabetes, and metabolic risk abnormalities.

Among the 192 no-NAFLD subjects without evidence of hepatic steatosis on ultrasonography, 143 (74.5%) were also excluded from the MAFLD patients’ group, while 49 (25.5%) were reclassified as MAFLD mainly for a FLI > 60 and of overweight/obesity or metabolic risk alterations. Ultimately, 816 (82.7%) patients were classified as MAFLD and 171 (17.3%) as no-MAFLD (Figure 2). Among NAFLD patients, 94.9% had overweight/obesity or diabetes as diagnostic criteria for MAFLD in addition to the detection of liver steatosis, while 5.1% had the presence of at least two metabolic risk abnormalities, in the absence of overweight/obesity and diabetes (lean MAFLD).

Table 2 reports some clinical and biochemical data of subjects with and without NAFLD reclassified into MAFLD and non-MAFLD, respectively. As reported in the table, 49 subjects with no NAFLD reclassified as MAFLD were more obese (BMI 30.4 ± 4.1 vs. 25.6 ± 2.9; *p* < 0.001) and had significantly higher levels of serum liver enzymes as compared to those who were classified as no-MAFLD.

Overall, 816 patients were classified as MAFLD after the application of the new criteria. However, in the analysis of original groups, these were significantly different regarding age, BMI, diabetes, MetS, HOMA-IR, liver transaminases levels, and atherogenic dyslipidemia (Table 2).

NAFLD subjects reclassified as no-MAFLD (*n* = 28) were younger, had no diabetes nor metabolic syndrome, and presented higher FIB-4 and APRI scores as compared to those reclassified as MAFLD.

Finally, 154 patients were lean (BMI < 25 kg/m^2^). Of these, 92 patients (59.7%) had NAFLD and only 66 (42.9%) had MAFLD.

## 4. Discussion

There are few papers that have investigated the reclassification effect of the new MAFLD criteria on patients currently considered as NAFLD.

We applied these criteria to a large prospective cohort study designed to investigate clinical outcomes in NAFLD patients (Plinio study). Overall, in the “Plinio study”, we observed a good agreement in classifying patients with the diagnostic criteria for NAFLD and those for MAFLD, although not all individuals with NAFLD had MAFLD and vice versa. The only feature differentiating MAFLD from NAFLD was a higher prevalence of overweight (BMI > 25 kg/m^2^) in the first. This result could allow obesity to be considered as the main metabolic characteristic of patients with MAFLD.

These findings are in keeping with the retrospective study performed by Lin at al. [31] showing concordance between MAFLD and NAFLD diagnostic criteria in the Third National Health and Nutrition Examination Survey (NHANES) 1988–1994 database. In addition, they found that MAFLD patients had higher liver enzymes and more glucose and lipid metabolism-related disorders.

Most patients with formerly named NAFLD (96.5%) enrolled in the “Plinio study” were confirmed to have MAFLD. Almost all (94.9%) were overweight and/or diabetic. The remaining (5.1%) of the NAFLD patients reclassified as MAFLD had neither overweight/obesity nor diabetes, i.e., lean MAFLD, but a combination of two or more metabolic risk abnormalities.

However, the analysis of the characteristics of the original groups of patients reclassified as MAFLD indicates some important clinical differences regarding age, BMI, diabetes, MetS, HOMA-IR, liver transaminases levels, and atherogenic dyslipidemia, which were less prevalent in patients belonging to the original no-NAFLD group. This evidence should be carefully considered as it may indicate that under the new MAFLD definition, there may be patients with very different clinical and biochemical features grouped under the same clinical entity, with no clear advantage compared to the current NAFLD definition.

On the other hand, our results demonstrate that new MAFLD criteria are more inclusive and reduce the unexplained form of lean NAFLD identifying the presence of metabolic risk factors in these patients. Among 68 lean NAFLD patients, 40 were defined as MAFLD and 28 as no-MAFLD. Lean NAFLD is usually defined as NAFLD that develops in patients with a body mass index (BMI) < 25 kg/m^2^. In these subjects, given the absence of classical risk factors, steatosis is often underrecognized. The prevalence of lean NAFLD is higher in some areas of the world, especially in the rural areas of Asian countries [32]. Recent publications show that lean NAFLD is not a simple benign condition [33,34]. In fact, in a large series of patients with biopsy-proven NAFLD, those with lean NAFLD were more likely to develop severe liver disease; older age, fibrosis stage, and hypertension were prognostic indicators for mortality [35]. Moreover, in a large follow-up study using U.S. population data from the NHANES III database, the presence of NAFLD in lean individuals was independently associated with increased risk of all-cause and cardiovascular mortality.

On the contrary, a minority of NAFLD subjects (3.5%), despite having fatty liver disease at ultrasound, were not overweight or obese and did not have diabetes or metabolic risk abnormalities, and therefore, could not be classified as MAFLD, i.e., metabolic healthy NAFLD. More specifically, none of these lean subjects had metabolic syndrome and very few had insulin resistance, increased waist circumference, high blood pressure, and dyslipidemia. Interestingly, in this group, the prevalence of values indicative for severe fibrosis of FIB-4, NFS, and APRI- was higher than that observed in subjects reclassified as MAFLD. These patients stand for a new challenge for understanding liver steatosis pathophysiology and might be underdiagnosed with new criteria. In fact, based on our results, MAFLD criteria, in comparison to NAFLD ones, had a lower ability to detect the disease (42.9% vs. 59.7%) in lean patients (BMI < 25 kg/m^2^).

In this study, 49 subjects without hepatic steatosis on ultrasound were classified as MAFLD. Most of these patients were obese, but the prevalence of those with diabetes and the metabolic syndrome was much lower than that seen in NAFLD patients who were reclassified as MAFLD. Similarly, in the same subjects, there was also less insulin resistance, a lower prevalence of dyslipidemia, and lower ALT values. In these patients, according to the new MAFLD diagnostic criteria, the diagnosis of steatosis was made based on FLI values >60.

US may have some limitations in the detection of liver steatosis due to the low sensitivity when compared to liver biopsy. In fact, US gives a negative response when less than 20–30% of hepatocytes have steatosis.

### Study Implications

While the definition of NAFLD was based on the absence of other liver diseases, that of MAFLD incorporates ‘positive diagnostic criteria’, and ensures that MAFLD is a clear, distinct clinical entity based on the presence of a metabolic dysfunction. Our data show that there is a substantial overlap between the definitions of NAFLD and MAFLD in most patients, but there are some specific subgroups of patients, such as those with lower BMI, that need more attention and probably additional diagnostic criteria (i.e., genetic characterization), as most of them cannot be defined as MAFLD with the proposed criteria, despite a US-proved liver steatosis. Furthermore, the prognostic value of this new classification needs to be explored and compared to the NAFLD one.

The study has some limitations. In keeping with the “Plinio study” exclusion criteria, none of the patients classified as NAFLD presented excess alcohol consumption and/or viral hepatitis, although these are no longer exclusion criteria for MAFLD diagnosis. A further limitation is not having included the high-sensitivity C reactive protein serum values (hs-PCR) among the diagnostic criteria for MAFLD, since h-PCR was not assessed in the Plinio study. However, this biomarker is highly non-specific and not required for the diagnosis of NAFLD.

## 5. Conclusions

Our results show a substantial overlap between NAFLD and MAFLD definitions, but some important differences may be present especially in patients with non-metabolic fatty liver disease (i.e., lean NAFLD patients).

## Figures and Tables

**Table 1:** Clinical and biochemical characteristics of subjects according to the diagnosis of NAFLD and MAFLD.

	MAFLD (*n* = 816)	No-MAFLD (*n* = 171)	*p*	NAFLD (*n* = 795)	No-NAFLD (*n* = 192)	*p*	*p* (NAFLD vs. MAFLD)
Age (years)	56.0 ± 12.3	57.3 ± 13.7	0.205	55.8 ± 12.3	58.0 ± 13.5	0.033	0.763
Women (%)	38.6	45.6	0.089	39.1	42.7	0.362	0.832
BMI (kg/$m^{2}$)	30.7 ± 5.0	25.2± 2.9	0.000	30.5 ± 5.2	26.8 ± 3.8	0.000	0.319
BMI ≥ 25 kg/$m^{2}$ (%)	92.0	49.1	0.000	88.6	68.2	0.000	0.018
Diabetes (%)	29.2	10.5	0.000	29.1	13.0	0.000	0.961
Metabolic Syndrome (%)	60.0	18.2	0.000	59.5	25.1	0.000	0.839
HOMA-IR > 2.5 (%)	66.9	18.7	0.000	67.2	22.9	0.000	0.912
Prior MACCE (%)	6.0	7.6	0.434	5.9	7.8	0.330	0.937
AST (UI/L)	21.0 [17.0–28.0]	19.0 [16.0–23.0]	0.000	21.0 [17.0–28.0]	18.0 [16.0–20.0]	0.000	0.569
ALT (UI/L)	27.0 [19.0–40.0]	17.0 [14.0–24.0]	0.000	27.0 [19.0–42.0]	18.0 [14.0–24.0]	0.000	0.705
GGT (UI/L)	27.0 [17.0–42.0]	18.0 [12.0–23.0]	0.000	26.0 [17.0–42.0]	18.0 [13.0–26.0]	0.000	0.778
FIB-4 > 2.67 (%)	2.6	3.4	0.588	2.6	3.1	0.713	0.964
APRI > 0.7 (%)	5.7	3.0	0.187	5.8	2.7	0.115	0.932
APRI > 1.0 (%)	2.3	1.3	0.448	2.6	0.0	0.037	0.704
High waist circumference (%)	82.5	41.2	0.000	80.2	55.0	0.000	0.246
High blood pressure (%)	73.6	57.6	0.000	72.4	64.4	0.028	0.861
Low HDL (%)	39.6	12.4	0.000	39.5	15.4	0.000	0.964
High triglycerides (%)	42.5	11.2	0.000	42.4	14.8	0.000	0.962
LDL-cholesterol (mg/dL)	115.0 [85.8–139.0]	116.0 [91.5–141.0]	0.548	115.5 [87.0–141.7]	116.0 [91.3–141.0]	0.744	0.986
Statin therapy (%)	39.6	47.6	0.055	39.4	47.6	0.038	0.959
Diabetes non-insulin therapy (%)	29.4	9.4	0.000	29.3	12.0	0.000	0.964
Insulin therapy (%)	2.8	1.2	0.212	2.9	1.0	0.143	0.929
Antiplatelet drugs (%)	15.4	17.4	0.493	15.2	18.2	0.305	0.902
Blood pressure medications (%)	61.8	53.2	0.038	61.3	56.3	0.203	0.834
Mediterranean Diet Score > 5 (%)	36.5	51.5	0.001	36.5	49.3	0.004	0.991

**Table 2:** Characteristics of patients with and without NAFLD reclassified into MAFLD and no-MAFLD, respectively.

	NAFLD to MAFLD (*n* = 767)	NAFLD to No-MAFLD (*n* = 28)	No-NAFLD to MAFLD (*n* = 49)	No-NAFLD to No-MAFLD (*n* = 143)	*p* all	*p* 3 vs. 4	*p* 1 vs. 3
Age (years)	56.0 ± 12.3	49.1 ± 10.8	55.0 ± 12.7	58.9 ± 13.6	0.001	0.196	0.035
Women (%)	38.7	50.0	40.5	45.2	0.335	0.403	0.782
BMI (kg/$m^{2}$)	30.7 ± 5.0	22.9 ± 1.7	30.4 ± 4.1	25.6 ± 2.9	0.000	0.000	0.000
BMI ≥ 25 kg/$m^{2}$ (%)	91.8	-	95.9	58.7	0.000	0.000	0.300
Diabetes (%)	30.1	-	14,3	12.6	0.000	0.760	0.018
Metabolic Syndrome (%)	61.7	-	34.7	21.8	0.000	0.073	0.000
HOMA-IR > 2.5 (%)	69.4	7.1	28.6	21.0	0.000	0.275	0.000
Prior MACCE (%)	6.1	0	4.1	9.1	0.238	0.259	0.559
AST (UI/L)	21.0 [17.0–28.0]	21.0 [17.0–27.0]	19.0 [17.0–21.0]	18.0 [16.0–22.0]	0.000	0.651	0.004
ALT (UI/L)	27.0 [19.0–42.0]	25.0 [17.0–43.0]	20.0 [17.0–27.0]	16.0 [13.0–22.0]	0.000	0.001	0.001
GGT (UI/L)	27.0 [17.0–42.0]	18.0 [13.0–34.0]	24.0 [15.0–51.0]	17.0 [12.0–22.5]	0.000	0.000	0.617
FIB-4 > 2.67 (%)	2.4	7.1	5.1	2.5	0.362	0.408	0.300
APRI > 0.7 (%)	5.8	8.3	5.1	1.8	0.328	0.277	0.869
APRI > 1.0 (%)	2.4	7.2	0	0	0.064	-	0.314
High waist circumference (%)	82.6	14.3	79.6	46.5	0.000	0.000	0.586
High blood pressure (%)	74.0	28.6	67.3	63.4	0.000	0.617	0.302
Low HDL (%)	40.8	3.6	19.6	14.1	0.000	0.371	0.004
High triglycerides (%)	43.9	3.6	21.3	12.7	0.000	0.160	0.002
LDL-cholesterol (mg/dL)	116.0 [91.7–140.4]	115.0 [85.5–150.7]	116.5 [89.2–153.2]	115.0 [85.8–139.0]	0.901	0.486	0.689
Statin therapy (%)	39.7	30.8	38.8	50.7	0.0.63	0.149	0.900
Diabetes non-insulin therapy (%)	30.4	0	14.3	11.2	0.000	0.565	0.017
Insulin therapy (%)	3.0	0	0	1.4	0.331	0.405	0.219
Antiplatelet drugs (%)	15.5	7.1	14.3	15.5	0.356	0.407	0.817
Blood pressure medications (%)	62.7	21.4	46.9	59.4	0.000	0.128	0.028
Mediterranean Diet Score > 5 (%)	36.3	42.3	39.5	53.3	0.010	0.127	0.670

## Data Availability

The data presented in this study are available on request from the corresponding author. The data are not publicly available due to privacy.

## References

[1] Ludwig J., Viggiano T.R., McGill D.B., Oh B.J. (1980). Nonalcoholic steatohepatitis: Mayo Clinic experiences with a hitherto unnamed disease. Mayo Clin. Proc..

[2] Cortez-Pinto H., Camilo M.E., Baptista A., De Oliveira A.G., De Moura M.C. (1999). Non-alcoholic fatty liver: Another feature of the metabolic syndrome?. Clin. Nutr..

[3] Schaffner F., Thaler H. (1986). Nonalcoholic fatty liver disease. Prog Liver Dis..

[4] Matteoni C.A., Younossi Z.M., Gramlich T., Boparai N., Liu Y.C., McCullough A.J. (1999). Nonalcoholic fatty liver disease: A spectrum of clinical and pathological severity. Gastroenterology.

[5] European Association for the Study of The Liver, European Association for the Study of Diabetes (2016). EASL-EASD-EASO Clinical Practice Guidelines for the management of non-alcoholic fatty liver disease. J. Hepatol..

[6] Day C.P., James O.F. (1998). Steatohepatitis: A tale of two “hits”?. Gastroenterology.

[7] Polimeni L., Del Ben M., Baratta F., Perri L., Albanese F., Pastori D., Violi F., Angelico F. (2015). Oxidative stress: New insights on the association of non-alcoholic fatty liver disease and atherosclerosis. World J. Hepatol..

[8] Tilg H., Moschen A.R. (2010). Evolution of inflammation in nonalcoholic fatty liver disease: The multiple parallel hits hypothesis. Hepatology.

[9] Baratta F., Pastori D., Bartimoccia S., Cammisotto V., Cocomello N., Colantoni A., Nocella C., Carnevale R., Ferro D., Angelico F. (2020). Poor Adherence to Mediterranean Diet and Serum Lipopolysaccharide are Associated with Oxidative Stress in Patients with Non-Alcoholic Fatty Liver Disease. Nutrients.

[10] Ferro D., Baratta F., Pastori D., Cocomello N., Colantoni A., Angelico F., Del Ben M. (2020). New Insights into the Pathogenesis of Non-Alcoholic Fatty Liver Disease: Gut-Derived Lipopolysaccharides and Oxidative Stress. Nutrients.

[11] Carpino G., Del Ben M., Pastori D., Carnevale R., Baratta F., Overi D., Francis H., Cardinale V., Onori P., Safarikia S. (2020). Increased liver localization of lipopolysaccharides in human and experimental non-alcoholic fatty liver disease. Hepatology.

[12] Del Ben M., Polimeni L., Brancorsini M., Di Costanzo A., D’Erasmo L., Baratta F., Loffredo L., Pastori D., Pignatelli P., Violi F. (2014). Non-alcoholic fatty liver disease, metabolic syndrome and patatin-like phospholipase domain-containing protein3 gene variants. Eur. J. Intern. Med..

[13] Di Costanzo A., Belardinilli F., Bailetti D., Sponziello M., D’Erasmo L., Polimeni L., Baratta F., Pastori D., Ceci F., Montali A. (2018). Evaluation of Polygenic Determinants of Non-Alcoholic Fatty Liver Disease (NAFLD) By a Candidate Genes Resequencing Strategy. Sci. Rep..

[14] Carpino G., Pastori D., Baratta F., Overi D., Labbadia G., Polimeni L., Di Costanzo A., Pannitteri G., Carnevale R., Del Ben M. (2017). PNPLA3 variant and portal/periportal histological pattern in patients with biopsy-proven non-alcoholic fatty liver disease: A possible role for oxidative stress. Sci. Rep..

[15] Di Costanzo A., D’Erasmo L., Polimeni L., Baratta F., Coletta P., Di Martino M., Loffredo L., Perri L., Ceci F., Montali A. (2017). Non-alcoholic fatty liver disease and subclinical atherosclerosis: A comparison of metabolically- versus genetically-driven excess fat hepatic storage. Atherosclerosis.

[16] Eslam M., Sanyal A.J., George J., Panel I.C. (2020). MAFLD: A Consensus-Driven Proposed Nomenclature for Metabolic Associated Fatty Liver Disease. Gastroenterology.

[17] Eslam M., Newsome P.N., Sarin S.K., Anstee Q.M., Targher G., Romero-Gomez M., Zelber-Sagi S., Wai-Sun Wong V., Dufour J.F., Schattenberg J.M. (2020). A new definition for metabolic dysfunction-associated fatty liver disease: An international expert consensus statement. J. Hepatol..

[18] Younossi Z.M., Rinella M.E., Sanyal A., Harrison S.A., Brunt E., Goodman Z., Cohen D.E., Loomba R. (2021). From NAFLD to MAFLD: Implications of a premature change in terminology. Hepatology.

[19] Bush K., Kivlahan D.R., McDonell M.B., Fihn S.D., Bradley K.A. (1998). The AUDIT alcohol consumption questions (AUDIT-C): An effective brief screening test for problem drinking. Ambulatory Care Quality Improvement Project (ACQUIP). Alcohol Use Disorders Identification Test. Arch. Intern. Med..

[20] Kim M.Y., Jeong W.K., Baik S.K. (2014). Invasive and non-invasive diagnosis of cirrhosis and portal hypertension. World J. Gastroenterol..

[21] Grundy S.M., Cleeman J.I., Daniels S.R., Donato K.A., Eckel R.H., Franklin B.A., Gordon D.J., Krauss R.M., Savage P.J., Smith S.C. (2005). Diagnosis and management of the metabolic syndrome: An American Heart Association/National Heart, Lung, and Blood Institute Scientific Statement. Circulation.

[22] American Diabetes Association (2020). 2. Classification and Diagnosis of Diabetes: Standards of Medical Care in Diabetes—2020. Diabetes Care.

[23] Mach F., Baigent C., Catapano A.L., Koskinas K.C., Casula M., Badimon L., Chapman M.J., De Backer G.G., Delgado V., Ference B.A. (2020). 2019 ESC/EAS Guidelines for the management of dyslipidaemias: Lipid modification to reduce cardiovascular risk. Eur. Heart J..

[24] Hindricks G., Potpara T., Dagres N., Arbelo E., Bax J.J., Blomström-Lundqvist C., Boriani G., Castella M., Dan G.A., Dilaveris P.E. (2021). 2020 ESC Guidelines for the diagnosis and management of atrial fibrillation developed in collaboration with the European Association for Cardio-Thoracic Surgery (EACTS): The Task Force for the diagnosis and management of atrial fibrillation of the European Society of Cardiology (ESC) Developed with the special contribution of the European Heart Rhythm Association (EHRA) of the ESC. Eur. Heart J..

[25] Williams B., Mancia G., Spiering W., Agabiti Rosei E., Azizi M., Burnier M., Clement D., Coca A., De Simone G., Dominiczak A. (2018). 2018 Practice Guidelines for the management of arterial hypertension of the European Society of Hypertension and the European Society of Cardiology: ESH/ESC Task Force for the Management of Arterial Hypertension. J. Hypertens..

[26] Hamaguchi M., Kojima T., Itoh Y., Harano Y., Fujii K., Nakajima T., Kato T., Takeda N., Okuda J., Ida K. (2007). The severity of ultrasonographic findings in nonalcoholic fatty liver disease reflects the metabolic syndrome and visceral fat accumulation. Am. J. Gastroenterol..

[27] Bedogni G., Bellentani S., Miglioli L., Masutti F., Passalacqua M., Castiglione A., Tiribelli C. (2006). The Fatty Liver Index: A simple and accurate predictor of hepatic steatosis in the general population. BMC Gastroenterol..

[28] Shah A.G., Lydecker A., Murray K., Tetri B.N., Contos M.J., Sanyal A.J. (2009). Comparison of noninvasive markers of fibrosis in patients with nonalcoholic fatty liver disease. Clin. Gastroenterol. Hepatol..

[29] Sterling R.K., Lissen E., Clumeck N., Sola R., Correa M.C., Montaner J., Sulkowski M.S., Torriani F.J., Dieterich D.T., Thomas D.L. (2006). Development of a simple noninvasive index to predict significant fibrosis in patients with HIV/HCV coinfection. Hepatology.

[30] Bourliere M., Penaranda G., Renou C., Botta-Fridlund D., Tran A., Portal I., Lecomte L., Castellani P., Rosenthal-Allieri M.A., Gerolami R. (2006). Validation and comparison of indexes for fibrosis and cirrhosis prediction in chronic hepatitis C patients: Proposal for a pragmatic approach classification without liver biopsies. J. Viral Hepat..

[31] Lin S., Huang J., Wang M., Kumar R., Liu Y., Liu S., Wu Y., Wang X., Zhu Y. (2020). Comparison of MAFLD and NAFLD diagnostic criteria in real world. Liver Int..

[32] Younossi Z., Anstee Q.M., Marietti M., Hardy T., Henry L., Eslam M., George J., Bugianesi E. (2018). Global burden of NAFLD and NASH: Trends, predictions, risk factors and prevention. Nat. Rev. Gastroenterol. Hepatol..

[33] Chrysavgis L., Ztriva E., Protopapas A., Tziomalos K., Cholongitas E. (2020). Nonalcoholic fatty liver disease in lean subjects: Prognosis, outcomes and management. World J. Gastroenterol..

[34] VanWagner L.B., Armstrong M.J. (2018). Lean NAFLD: A not so benign condition?. Hepatol. Commun..

[35] Hagström H., Nasr P., Ekstedt M., Hammar U., Stål P., Hultcrantz R., Kechagias S. (2018). Risk for development of severe liver disease in lean patients with nonalcoholic fatty liver disease: A long-term follow-up study. Hepatol. Commun..

